# Reduced native right ventricular T_1 _in Anderson-Fabry disease as compared to patients with pulmonary hypertension

**DOI:** 10.1186/1532-429X-16-S1-P2

**Published:** 2014-01-16

**Authors:** Joseph J Pagano, Kelvin Chow, Aneal Khan, Evangelos Michelakis, Ian Paterson, Gavin Y Oudit, Richard B Thompson

**Affiliations:** 1 Biomedical Engineering, University of Alberta, Edmonton, Alberta, Canada; 2 Medicine, University of Alberta, Edmonton, Alberta, Canada; 3 Medical Genetics, University of Calgary, Calgary, Alberta, Canada

## Background

Recently, native (non-contrast) T_1 _mapping has demonstrated low left ventricular myocardial values in patients with Anderson-Fabry disease (FD)[1,2], potentially due to accumulation of glycosphingolipids. Autopsy studies have shown biventricular involvement, however quantitative T_1 _imaging of the right ventricle (RV) has not been performed.

## Methods

In images from 20 subjects with FD and 7 subjects with pulmonary hypertension (PH), the inferior RV wall was assessed for the appearance of hypertrophy, to identify subjects with sufficient wall thickness for T_1 _analysis. Images were acquired on 1.5T Siemens systems (Sonata and Avanto) using the SASHA T_1 _mapping method[3] for all studies: 70° flip angle, 1.31 ms echo time, 2.62 ms repetition time, 9 images with 85-995 ms saturation recovery times plus a non-saturated image, 8 mm slice thickness, 360 × 270 FOV, 192 × 108 acquisition matrix before interpolation, and 75% phase resolution. Either rate 2 parallel imaging (GRAPPA) or 6/8 partial Fourier was used for image acceleration. Regions of interest were drawn on the septum and inferior RV by two observers, avoiding the septal insertion point, fat and blood pool. Analysis was repeated 10 times per subject to evaluate T_1 _variability with ROI placement. Late gadolinium enhancement (LGE) and diastolic regional wall thickness (WT), measured on bSSFP cine images, were assessed at a comparable slice location.

## Results

**Figure 1:**
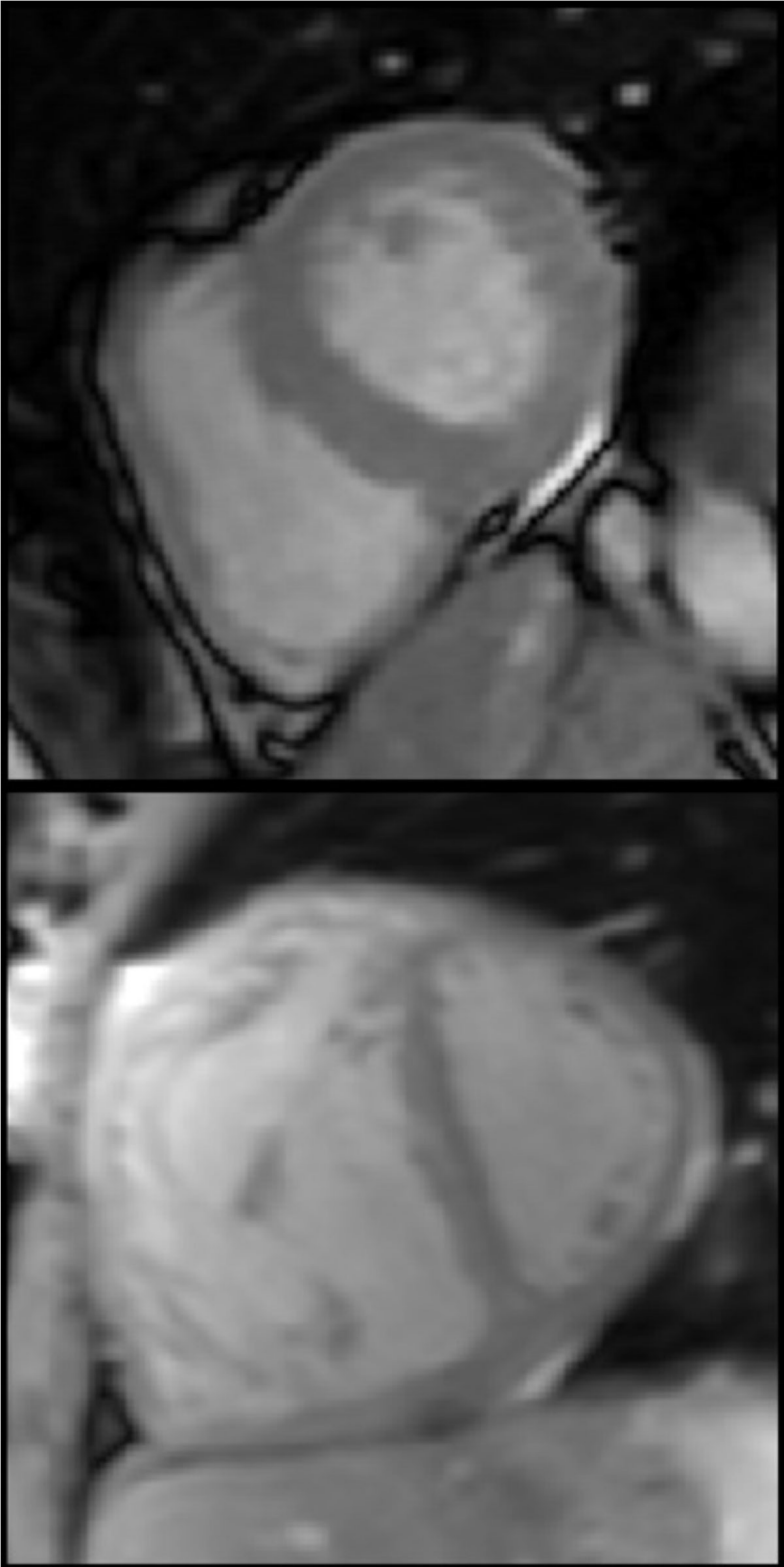
**Sample non-saturation SASHA images**: Anderson-Fabry disease (top) and pulmonary hypertension (bottom).

**Figure 2:**
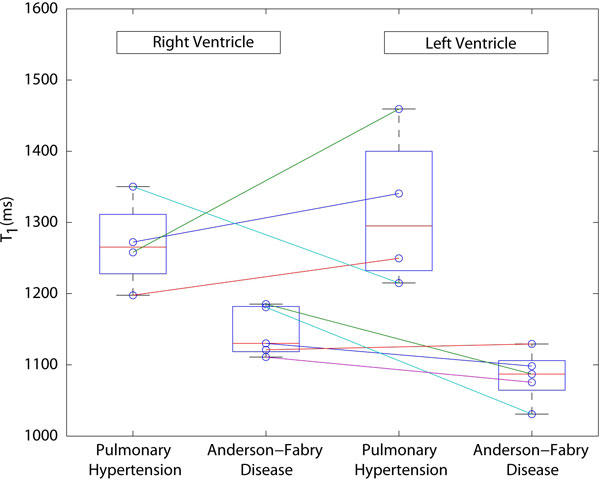
**Myocardial T_1 _values by ventricle and condition**. Circles and line segments denote values for a given individual, and * marks cases of septal late gadolinium enhancement.

Analysis was performed in 5 patients with FD and 4 patients with PH (WT_(LV-FD) _= 11.7 mm, WT_(RV-FD) _= 5.7 mm, WT_(LV-PH) _= 10.8 mm, WT_(RV-PH) _= 7.5 mm), as the remaining cases had inadequate hypertrophy and spatial resolution to proceed with analysis. Sample SASHA images are shown in Figure 1. Figure 2 shows T_1 _values for all subjects, demonstrating lower myocardial T_1 _in both the LV and RV for all FD subjects (p = 0.0143 for either ventricles). Interobserver agreement (coefficient of variation) was 4.0% and 2.41% for the RV and septum, respectively. No FD subjects were positive for LGE in the inferior RV or septum; however one was positive at the RV insertion point. No PH subjects were positive for LGE in the inferior wall; however 2 showed mid-wall septal enhancement at locations within ROIs and all were positive at the RV insertion site. However, as indicated in Figure 2, septal T_1 _values are similar in those positive (1340.7 & 1249.7 ms) and or negative (1459.4 & 1215.0 ms) for LGE.

## Conclusions

In FD patients with RV hypertrophy, both RV and LV native T_1 _values are reduced as compared to patients with PH and RV hypertrophy. Significant improvement in spatial resolution is required for T_1 _mapping of the normal right ventricle to establish healthy native RV T_1 _values.

## Funding

The authors acknowledge financial support from CIHR, AIHS, WCHRI.

## References

[B1] Thompson Circ Cardiovasc Imaging 2013

[B2] Sado Circ Cardiovasc Imaging 2013

[B3] Chow MRM 2013

